# Post-encephalitic epilepsy in patients with acute encephalopathy with biphasic seizures and late reduced diffusion

**DOI:** 10.3389/fneur.2025.1568566

**Published:** 2025-07-22

**Authors:** Kota Nagai, Go Kawano, Hirotaka Sakaguchi, Takaoki Yokochi, Nobuoki Eshima, Toyojiro Matsuishi

**Affiliations:** ^1^Department of Paediatrics, St Mary’s Hospital, Fukuoka, Japan; ^2^Department of Paediatrics and Child Health, Kurume University School of Medicine, Fukuoka, Japan; ^3^Research Centre for Children and Research Centre for Rett Syndrome, St Mary’s Hospital, Fukuoka, Japan; ^4^Division of Gene Therapy and Regenerative Medicine, Cognitive and Molecular Research Institute of Brain Diseases, Kurume University School of Medicine, Fukuoka, Japan

**Keywords:** acute encephalopathy with biphasic seizures and late reduced diffusion, postencephalic epilepsy, early seizure, late seizures, Tada score

## Abstract

**Introduction:**

Acute encephalopathy with biphasic seizures and late reduced diffusion (AESD) is a prevalent form of acute infection-triggered encephalopathy in children, excluding the unclassified type. It is marked by febrile seizures, referred to as early seizures and subsequent late seizures, along with a reduction in the apparent diffusion coefficient in cortical or subcortical white matter. AESD frequently results in neurological sequelae, including post-encephalitic epilepsy (PEE).

**Methods:**

This retrospective study examined the incidence of PEE and investigated whether therapeutic hypothermia reduced the risk of PEE in 45 patients with AESD treated at St Mary’s Hospital between July 2008 and April 2024. Patients whose Glasgow Coma Scale score was >8 and who had mild clinical symptoms, as judged by the attending physician, between July 2008 and December 2020, did not undergo therapeutic hypothermia. However, all patients with AESD underwent therapeutic hypothermia during the period between January 2021 and April 2024. There were 11, 24, and 10 patients in the Early-Hypo, Late-Hypo, and Non-Hypo groups, respectively.

**Results:**

The prevalence of PEE among patients with AESD was 26.7% (12 out of 45 patients), with a median observation period of 75 months (IQR 37–98 months, range 20–182 months). Univariate analysis revealed statistically significant differences between patients with and without PEE in several factors, including Glasgow Coma Scale scores between 12 and 24 h after early seizures, and the number of patients with AESD with biphasic course, Tada scores, and the Pediatric Cerebral Performance Category at 6 months post-onset. Multivariate logistic regression analysis identified higher Tada scores as an independent risk factor for developing PEE, and the treatment options of Early-Hypo or Late-Hypo significantly reduced the risk of PEE.

**Conclusion:**

While further prospective studies with larger cohorts are warranted, this study highlights the association between higher Tada scores and an increased risk of PEE in patients with AESD, and the treatment options of Early-Hypo or Late-Hypo significantly reduced the risk of PEE.

## 1 Introduction

In Japan, acute encephalopathy with biphasic seizures and late reduced diffusion (AESD) is the most common form of acute encephalopathy in children, with the exception of the unclassified type and a subtype of acute infection-triggered encephalopathy, accounting for 34% of all cases (1). It is marked by febrile seizures, referred to as early seizures (ESs), and subsequent late seizures (LSs), along with a reduced apparent diffusion coefficient in the cortical or subcortical white matter, often described as a bright tree appearance (2). The Japanese guideline for diagnosing and treating acute encephalopathy in childhood, issued by the Japanese Society of Child Neurology Committee in 2023, does not fully endorse hypothermia/normothermia therapy due to the absence of clinical randomized controlled trials (3). However, targeted temperature management is anticipated to reduce the risk of neurological sequelae based on robust preclinical and clinical neuroprotective evidence in encephalopathy following perinatal asphyxia and adult cardiac arrest (3–5). Indirect beneficial evidence reported by Sakata et al. suggests that the earlier therapeutic hypothermia is initiated in patients with AESD, the better the outcome (6). The prognosis ranges from normal or mild intellectual disability to severe psychomotor disability or death (1).

Post-encephalitic epilepsy (PEE) is one of the neurological sequelae of acute encephalitis or encephalopathy syndromes. The prevalence of epilepsy following central nervous system infections has been reported to range from 10 to 20% (7–9). The time interval between acute encephalitis and the onset of PEE varies widely, from 0 to 95 months, with 80% of cases developing PEE within the first 6 months (8).

In patients with AESD, the prevalence of PEE is notably higher, reported to range from 22 to 45.5%, exceeding that observed in epilepsy following other central nervous system infections. The time interval between acute encephalopathy and the onset of PEE is comparable, with a median of 2–14 months and a range of 0.8–48 months (10–13). Epileptic spasms, startle seizures, and focal seizures are common in patients with AESD with PEE, and 40–66.7% of these cases are reported as intractable (10–12). Identified risk factors for PEE include elevated aspartate aminotransferase (AST) and/or alanine aminotransferase (ALT) levels at onset, coma or involuntary movements during the AESD course, broader areas of reduced diffusion in the posterior lobes at onset, and poorer outcomes at follow-up (10–12).

To examine the incidence of PEE and investigate whether therapeutic hypothermia reduced the risk of PEE, we performed a retrospective study on patients with AESD, comparing those who developed PEE to those who did not.

## 2 Materials and methods

### 2.1 Survey method

We enrolled patients with AESD admitted at St Mary’s Hospital during the study period between July 2008 and April 2024. The diagnosis of AESD in this study was based on the diagnostic criteria for definite and possible AESD reported by Sakuma et al. (14). The diagnostic criteria for AESD included the following: (1) febrile illness preceding or concurrent with the onset of neurological manifestations, (2) a clinical presentation involving a decreased or altered level of consciousness, altered mental status, lethargy, or personality change persisting for > 24 h, and (3) restricted diffusion on magnetic resonance imaging (MRI) in the subcortical white matter (bright tree appearance) observed between days 3 and 14 after the onset of neurological symptoms. Patients in whom LSs could not be distinguished from ESs or were masked by disease severity, aggressive antiseizure therapy, or sedatives were diagnosed as having possible AESD. Additionally, in cases where MRI was not performed in the acute phase due to the patient’s critical condition, findings such as subcortical hyperintensity on T2/fluid-attenuated inversion recovery imaging or decreased blood flow on single photon emission computed tomography (SPECT) or arterial spin labeling were considered supportive of a possible AESD diagnosis (14).

**Figure 1:**
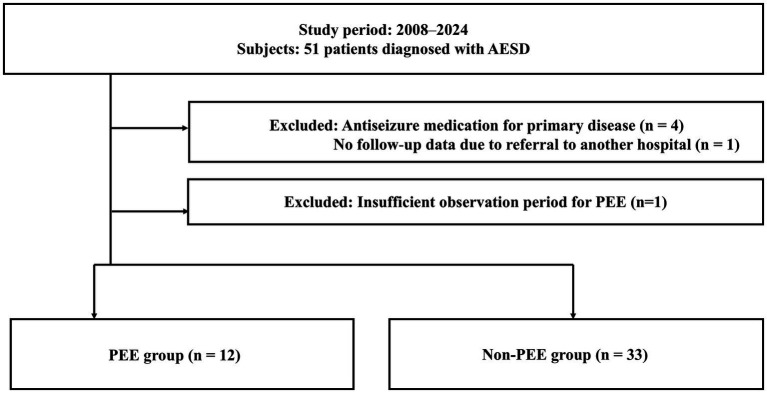
Study sample. Fifty-one patients with AESD were admitted to our facility between July 2008 and April 2024. Four patients on antiseizure medication (ASM) prior to AESD onset for epilepsy associated with primary diseases (developmental disorder due to sepsis during the perinatal period, muscle -eye -brain syndrome, PIGO deficiency, or brain tumor), and one without follow-up data due to referral to another hospital, were excluded from this study. In total, 45 patients with AESD who underwent therapeutic hypothermia were included, of whom 12 patients had PEE and 33 patients did not have PEE.

A total of 51 patients with AESD who were admitted to our facility between July 2008 and April 2024 were considered for the study, and of these, six patients were excluded (Figure 1). Four of the six patients were already on antiseizure medications (ASMs) for epilepsy prior to the onset of AESD associated with primary conditions (developmental disorder due to perinatal sepsis, muscle-eye-brain syndrome, PIGO deficiency, and brain tumor). One patient lacked follow-up data after referral to another hospital. One patient was excluded because the observation period after the onset of AESD was 12 months, which was considered to be too short to categorize as the non-PEE group. The final analysis included 45 patients with AESD.

Of the patients included in this study, 29, 11, and 6 were part of our previous studies on the determinants of outcomes, frontal ataxia, and arterial spin labeling imaging findings in patients with AESD, respectively (6, 15, 16). Additionally, 13 patients were included in our ongoing project on remote ischaemic postconditioning (RIPoC) (UMIN000041484).

### 2.2 Therapeutic hypothermia

Of the 51 patients with AESD admitted to our facility between July 2008 and April 2024, 37 were admitted between July 2008 and December 2020, and 14 were admitted between January 2021 and April 2024.

Notably, 27 out of 37 patients admitted between July 2008 and December 2020 received therapeutic hypothermia. Throughout the study period, we provided therapeutic hypothermia for this patient group, following our institutional protocol for managing patients with consciousness disturbance (6). According to the protocol, therapeutic hypothermia should be available for patients with continuous consciousness disturbance and a haemodynamically stable condition. When a patient continuously has a GCS score < 9, therapeutic hypothermia can be applied. For a GCS score > 8, the decision to apply therapeutic hypothermia was based on the attending physicians’ judgment of the severity of the neurological symptoms of that patient. The remaining 10 patients were not cooled by the attending physicians due to mild clinical symptoms, such as high GCS score at 12–24 h after ESs or during LSs, lower Tada score, and absence of biphasic course, even though the MRI image showed AESD findings.

Conversely, all 14 patients admitted between January 2021 and April 2024 underwent therapeutic hypothermia. During this period, therapeutic hypothermia was applied when patients had persistent consciousness disturbance for more than 6 h and were later confirmed to have AESD through neuroimaging or just after when MRI findings revealed AESD even if the patient had mild symptoms after ES/ESs or during LSs.

Therapeutic hypothermia was administered using mattresses with a circulating temperature-adjustable water set (Medivance Arctic Sun®, Medivance Inc., Louisville, CO, USA) to maintain a core temperature of 33.5–35°C. These mattresses were applied to the ventral and/or dorsal trunk, and hypothermia was maintained for 72 h. Detailed methods for therapeutic hypothermia have been previously described (6). Based on the timing of therapeutic hypothermia initiation, patients were classified into three groups: those who commenced therapeutic hypothermia prior to the onset of LSs due to a worsening level of consciousness or persistently impaired consciousness (Early-Hypo group), those who initiated therapeutic hypothermia after the onset of LSs (Late-Hypo group), and those who were not cooled (Non-Hypo group).

### 2.3 PEE

We prescribed ASMs, levetiracetam (LEV) or carbamazepine (CBZ) to patients with AESD at our institution for 6 to 24 months after the acute phase to prevent subsequent febrile or afebrile seizures. This was because previous reports indicated that the time interval between acute encephalopathy and the development of PEE is within 12 months after onset in most cases (10–13).

PEE was defined, regardless of ASM prescription, as the occurrence of multiple unprovoked seizures following the cessation of continuous sedation for intensive care, typically around 14 d after early seizures, and requiring dose escalation of existing ASMs or the initiation of new ASMs regardless of the presence or absence of abnormal electroencephalography findings. This definition was adapted from the criteria used by Ichimiya et al. (10). The lower limit of the observation period for PEE was set at 20 months after the onset of AESD (10–13), and one patient was excluded from our study as described above.

Of the 45 patients included in this study, 44 had an observation period of more than 2 years and 3 months, and the remaining patient had an observation period of 20 months. Based on the presence of PEE during the observation period, the patients were classified into two groups: those who had PEE (the PEE group) and those who did not (the non-PEE group).

### 2.4 Data collection

Clinical variables were collected for each patient, including age, sex, and associated infections. The duration of ESs was defined as the time from the beginning of the seizure, which was recorded based on their parents’ or guardians’ observation in most of the cases, regardless of convulsive or non-convulsive seizure, until the clinical cessation of the seizure, which was usually achieved after introduction of an antiseizure medication in the hospital. When patients did not recover consciousness between seizures, it was considered a single persistent seizure. GCS scores were recorded between 12 and 24 h after the first seizure of ESs (GCS 12–24 h). Time intervals (in hours) were assessed, including the interval between the first seizure of ESs and LSs (Time_1st − 2nd_) in the Late-Hypo group, the interval between the first seizure of ESs and the initiation of therapeutic hypothermia (Time_1st-cooling_) in both Early-Hypo and Late-Hypo groups, and the interval between the first seizure of LSs and the initiation of therapeutic hypothermia (Time_2nd-cooling_) in the Late-Hypo group. Neuroimaging data included the distribution of brain lesions on MRI (hemisphere, bilateral frontal lobe, or other regions), the absence of diffuse lesions with injury around the perirolandic regions (central sparing) on MRI in bilateral hemispheres, and the presence of basal ganglia or thalamus lesions on MRI. Biochemical markers were evaluated, including AST, ALT, lactate dehydrogenase (LDH), blood urea nitrogen, creatinine, glucose, and platelet count at the time of ESs. Additional assessments included Tada and Yokochi scores after ESs (17, 18), treatment options, such as the use of intravenous methylprednisolone pulse therapy (30 mg/kg/dose for 3 d) or immunoglobulin therapy (1 g/kg/dose), and treatment groups (the Early-Hypo, Late-Hypo, or Non-Hypo group). Key outcomes included the presence of PEE, the duration (in months) between the onset of AESD and PEE occurrence, seizure semiology, and interictal electroencephalography findings around the time of PEE occurrence in PEE, and neurological outcomes.

The Tada score is a predictive tool for AESD in patients presenting with febrile seizures during the acute phase (17). Similarly, the Yokochi score is also a predictive measure for AESD designed to assess the risk of developing this condition (18). Neurological outcomes were evaluated at 6 months from the onset of AESD using the Pediatric Cerebral Performance Category Scale (PCPC). This scale classifies outcomes into six stages: 1, normal performance; 2, mild disability; 3, moderate disability; 4, severe disability; 5, persistent vegetative state; and 6, death (19).

Furthermore, the data of the most recent neurodevelopment or cognitive test at follow-up were collected. Events of hypotension, defined as systolic blood pressure below average values (−2SD for patients’ age) according to the Task Force on Blood Pressure Control in Children (20), were recorded irrespective of catecholamine use and requirement.

In the non-PEE group, no blood test was conducted just after ESs in a patient, and the LDH level was not checked after ESs in another patient.

All patients, except for five in the non-PEE group, were prescribed ASMs after the acute phase. In the non-PEE group, ASMs were used for a median duration of 27 months (IQR 18–34 months; range 6–56 months), including tapering periods. In two cases, ASMs (LEV or lacosamide) were used prophylactically (71 and 92 months) for febrile seizures at the time of investigation.

### 2.5 Statistical analyses

SPSS version 25 (IBM Corp., Armonk, NY, USA) was used for all statistical analyses except for Fisher’s exact binomial test with Bonferroni correction as the *post hoc* test for categorical and discrete variables, for which R version 3.6.2 (21)^1^ was used. First, differences between the PEE and non-PEE groups were evaluated. Clinical variables and treatment options were analyzed using the Mann–Whitney U test and Fisher’s exact test (two-tailed) for continuous variables and categorical variables, respectively. A *p*-value of < 0.05 was considered statistically significant. Second, an intergroup analysis between the treatment options of Early-Hypo, Late-Hypo, or Non-Hypo group, clinical variables, treatment options, and associated events mentioned above was examined using the Kruskal-Wallis test and Dunn-Bonferroni *post hoc* test for variables between the three groups. Clinical variables and treatment options were analyzed using the Mann–Whitney U test (two-tailed) for variables between two groups, followed by Fisher’s exact binomial test with Bonferroni correction as the post hoc test for categorical and discrete variables. Third, a multivariate logistic regression analysis was performed to identify factors associated with PEE occurrence within the study population.

This study was conducted with the approval of the Ethics Committee of St Mary’s Hospital (IRB number: 24–1,010).

## 3 Results

The prevalence of PEE among all patients with AESD was 26.7% (12 out of 45 patients), with a median observation period of 75 months (IQR 37–98 months, range 20–182 months).

### 3.1 Clinical characteristics of each treatment group and multiple comparison test

The clinical characteristics of each group are shown in Table 1 and Supplementary Table 1. There were 11, 24, and 10 patients in the Early-Hypo, Late-Hypo, and Non-Hypo groups, respectively. GCS 12–24 h of the Early-Hypo group was significantly worse than that of the Late-Hypo or Non-Hypo group (*p* < 0.001, *p* = 0.010, Supplementary Figure 1). The Tada score of the Early-Hypo group was significantly higher than that of the Late-Hypo group (*p* = 0.033, Supplementary Figure 2). The Late-Hypo group had a larger number of patients with AESD with a biphasic course than the No-Hypo group (*p* = 0.005). All three results were reflected by the indication of therapeutic hypothermia between July 2008 and December 2020, judged by the attending physicians based on the severer clinical symptoms, such as lower GCS 12–24 h or during LSs, higher Tada score, or presence of biphasic course, as described above. Additionally, the number of patients with AESD using catecholamine in the Non-Hypo group was significantly lower than that of the Early-Hypo or Late-Hypo groups (*p* = 0.009, *p* < 0.001), naturally because of the unnecessary use of continuous intravenous sedatives and/or therapeutic hypothermia, which often decrease the blood pressure necessitating catecholamine use. PCPC at 6 months after onset in the Early-Hypo group was significantly worse than that of the Late-Hypo group (*p* = 0.043, Supplementary Figure 3).

**Table 1:** Multiple comparison among patients in the Early-Hypo, Late-Hypo, and Non-Hypo groups (*n* = 46).

	Early-Hypo group			Late-Hypo group			Non-Hypo group			*p* value
	*n* = 11			*n* = 24			*n* = 10			
Age in months, median (IQR), *n*	15	(10-22)	11	16	(13-19)	24	26.5	(14–48)	10	0.075
Sex, Female, *n* (%)	8	(72.7)	11	9	(37.5)	24	5	(50)	10	0.181
GCS 12–24 h after ES/ESs	9	(7.5–11.5)	11	15	(13.5–15)	24	14.5	(13-15)	10	< 0.001*
AST, IU/L, median (IQR), *n*	49	(43–81.5)	11	47	(385–54)	24	39	(37–55)	9	0.283
ALT, IU/L, median (IQR), *n*	22	(13.5–38.5)	11	16	(12.5–27.5)	24	17	(12–24)	9	0.628
LDH, IU/L, median (IQR), *n*	347	(317.5–421.5)	11	344	(324.5–390)	23	331	(327–345)	9	0.409
Creatinine, mg/dL, median (IQR), *n*	0.31	(0.27–0.36)	11	0.30	(0.26–0.33)	24	0.32	(0.26–0.47)	9	0.741
Platelet, x10^3^/μL, median (IQR), *n*	28.1	(22.5–33.3)	11	26.8	(19.0–31.7)	24	23.6	(19.5–37.3)	9	0.780
Blood glucose, mg/dL, median (IQR), *n*	244	(168.5–271.5)	11	168	(132.5–256)	24	196	(126–239)	9	0.768
ES duration in min, median (IQR), *n*	50	(42.5–69)	11	42	(22–65.5)	24	10	(4–45)	9	0.063
Distribution of the lesion			11			24			10	0.1724
Hemisphere, *n* (%)	0	(0)		7	(29.2)		3	(30)		
Bilateral Frontal, *n* (%)	6	(54.5)		7	(29.2)		5	(50)		
Others, *n* (%)	5	(45.5)		10	(41.7)		2	(20)		
Central sparing, *n* (%)	10	(100)	10	23	(95.8)	24	10	(100)	10	1.000
Basal ganglia or thalamus lesion, *n* (%)	2	(20)	10	7	(29.2)	24	2	(20)	10	0.810
Tada score, median (IQR), *n*	5	(5–6.5)	11	3	(2-5)	23	3	(2-4)	9	0.021^†^
Yokochi score, median (IQR), *n*	4	(3-7)	11	3	(1.5–5)	23	1	(0–5)	9	0.062
Biphasic course, *n* (%)	N/A	N/A	N/A	24	(100)	24	6	(60)	10	0.005^‡^
Duration between ES/ESs and LSs (Time_1st-2nd_) in hours, median (IQR), *n*	N/A	N/A	N/A	88	(67.8–103.8)	24	108	(90–108)	5	0.222
Therapeutic options										
Steroid, *n* (%)	4	(36.4)	11	4	(16.7)	24	4	(40)	10	0.268
Intravenous immunoglobulin, *n* (%)	2	(18.2)	11	2	(8.3)	24	2	(20)	10	0.597
RIPoC	4	(36.4)	11	8	(33.3)	24	0	(0)	10	0.104
Time to cooling initiation from ES/ESs (Time_1st-cooling_) in hours, median (IQR), *n*	23.1	(16.7–33.5)	11	92.5	(79–113)	24	N/A	N/A	N/A	0.168
Time to cooling initiation from LSs (Time_2nd-cooling_) in hours, median (IQR), *n*	N/A	N/A	N/A	9.5	(6.5–12.5)	23	N/A	N/A	N/A	N/A
Associated events										
Hypotension, *n* (%)	4	(36.4)	11	10	(41.7)	24	1	(1)	10	0.211
Catecholamine use, *n* (%)	7	(63.6)	11	17	(70.8)	24	0	(0)	10	< 0.001^§^
PCPC at 6 months after onset	3	(2-3)	11	2	(1-3)	24	2	(2-2)	10	0.044^¶^
Prophylactic use of ASM, *n* (%)	10	(90.9)	11	23	(95.8)	24	7	(70)	10	0.053
PEE, *n* (%)	4	(36.4)	11	4	(16.7)	24	4	(40)	10	0.268
Duration from the onset of AESD to the development of PEE in months, median (IQR), *n*	2	(0.9–16.5)	4	5.0	(0.8–12)	4	12	(8.5–32)	4	0.269
Observation period in months, median (IQR), *n*	79	(36.5–103)	11	67.5	(36.5–94)	24	87.5	(74–117)	10	0.495

^*^The Early-Hypo vs the Late-Hypo group, Non-Hypo group (*p* < 0.001, *p* = 0.010 in Dunn-Bonferroni *Post hoc* test, respectively).

^†^The Early-Hypo vs the Late-Hypo group (*p* = 0.033 in Dunn-Bonferroni *Post hoc* test).

^‡^*p* < 0.05 in the Fisher’s exact test.

^§^The Non-Hypo vs the Early-Hypo, Late-Hypo group (*p* = 0.009, *p* < 0.001 in Fisher’s exact binomial test with Bonferroni correction, respectively).

^¶^The Early-Hypo vs the Late-Hypo group (*p* = 0.043 in Dunn-Bonferroni *Post hoc* test).

N/A, not applicable.

PEE was identified in 4 patients in each group (36.4, 16.7, and 40% in the Early-Hypo, Late-Hypo, and Non-Hypo groups, respectively). The duration from the onset of AESD to the development of PEE was assessed for each group. Although the duration of the Non-Hypo groups was relatively longer than other two groups, no significant difference was observed among three groups.

### 3.2 Clinical characteristics of the PEE and non-PEE groups, and univariate analysis between the groups

The clinical characteristics of each group (12 patients in the PEE group and 33 patients in the non-PEE group) are shown in Table 2 and Supplementary Table 2. The observation period of each group was a median of 87 months (IQR 38–94.5 months) and 74 months (IQR 37–108 months), respectively (Supplementary Figure 4). The median ages of the PEE and non-PEE groups were 15 months (IQR 10.5–29.5 months) and 16 months (IQR 13–21 months), respectively. No significant difference was observed between the two groups in therapeutic variables, including steroid or intravenous immunoglobulin use, treatment options (Early-Hypo, Late-Hypo, or Non-Hypo groups), Time_1st-2nd_ (Late-Hypo group), Time_1st-cooling_ (Early-Hypo and Late-Hypo groups), or Time_2nd-cooling_ (Late-Hypo group).

**Table 2:** Univariate analysis between PPE and non-PEE groups (*n* = 36).

	PEE group			Non-PEE group			*p* value
	*n* = 12			*n* = 33			
Age in months, median (IQR), *n*	15	(10.5–29.5)	12	16	(13-21)	33	0.790
Sex, Female, *n* (%)	7	(58.3)	12	15	(46.9)	32	0.514
GCS 12–24 h after ES/ESs, median (IQR), *n*	12	(10-14)	12	15	(11-15)	33	0.006*
AST, IU/L, median (IQR), *n*	57	(37–84)	12	43.5	(38.5–54)	32	0.255
ALT, IU/L, median (IQR), *n*	26	(12.5–39)	12	16	(13–23.5)	32	0.195
LDH, IU/L, median (IQR), *n*	364.5	(327–421.5)	12	340	(319.5–383)	32	0.289
Creatinine, mg/dL, median (IQR), *n*	0.32	(0.29–0.38)	12	0.28	(0.25–0.34)	32	0.169
Platelet, x10^3^/μL, median (IQR), *n*	32.0	(18.4–39.4)	12	26.6	(20.3–30.5)	32	0.328
Blood glucose, mg/dL, median (IQR), *n*	247.5	(134.5–297)	12	172	(132.5–247)	32	0.341
ES duration in min, median (IQR), *n*	50	(42–71.5)	12	40	(13.5–60)	32	0.245
Distribution of the lesion			12			32	0.571
Hemisphere, *n* (%)	3	(25)		7	(21.9)		
Bilateral Frontal, *n* (%)	6	(50)		12	(37.5)		
Others, *n* (%)	3	(25)		14	(43.8)		
Central sparing, *n* (%)	10	(90.9)	11	33	(100)	33	0.250
Basal ganglia or thalamus lesion, *n* (%)	5	(45.5)	11	6	(18.2)	33	0.250
Tada score, median (IQR), *n*	6	(5-6)	12	3	(2–4.5)	31	0.002*
Yokochi score, median (IQR), *n*	5.5	(2.5–6.5)	12	3	(1–4.5)	31	0.067
Biphasic course, *n* (%)	5	(50)	8	25	(75)	26	0.033
Duration between ES/ESs and LSs (Time_1st-2nd_) in hours, median (IQR), *n*	69	(64–80)	5	92.5	(83.8–110.5)	24	0.089
Therapeutic options							
Steroid, *n* (%)	3	(25)	12	9	(27.3)	33	1.000
Intravenous immunoglobulin, *n* (%)	3	(25)	12	3	(9.1)	33	0.319
RIPoC	4	(33.3)	12	8	(24.2)	33	0.705
Therapeutic hypothermia, *n* (%)	8	(66.7)	12	27	(81.8)	33	0.418
Treatment groups based on the timing of therapeutic hypothermia			12			33	0.268
Early-Hypo, *n* (%)	4	(33.3)		7	(21.2)		
Late-Hypo, *n* (%)	4	(33.3)		20	(60.6)		
Non-Hypo, *n* (%)	4	(33.3)		6	(18.2)		
Time to cooling initiation from ES/ESs (Time_1st-cooling_), in months, median (IQR), *n*	69	(24.6–87.8)	8	91.5	(45.5–107.5)	27	0.192
Time to cooling initiation from LSs (Time_2nd-cooling_), in months, median (IQR), *n*	8.3	(6.5–16.3)	4	9.5	(6–12.5)	19	0.845
Associated events							
Hypotension, *n* (%)	5	(41.7)	12	12	(36.4)	33	0.743
Catecholamine use, *n* (%)	5	(50)	12	18	(54.5)	33	1.000
PCPC at 6 months after onset, median (IQR), *n*	3	(2–3.5)	12	2	(1-2)	33	0.004*
Observation period in months, median (IQR), *n*	87	(38–94.5)	12	74	(37–108)	33	0.970

*p < 0.05.

Preceding infections were identified in five patients in the PEE group and 18 patients in the non-PEE group. Notably, among the five in the PEE group, ECHO virus 22 (*n* = 2), influenza A (*n* = 1), influenza B (*n* = 1), and *Streptococcus pneumoniae* meningitis (*n* = 1) were identified. In the non-PEE group, exanthem subitum (*n* = 6), influenza A (*n* = 3), varicella-zoster virus (*n* = 2), Coxsackievirus A4 (*n* = 2), adenovirus, human metapneumovirus, parvovirus B19, rotavirus, and respiratory syncytial virus were identified. Preceding infections were confirmed by at least one positive result from viral culture, antigen testing, reverse transcription PCR, or significantly elevated titres in paired serum samples. One patient in the PEE group was diagnosed with Kawasaki disease, and four in the non-PEE group were clinically diagnosed with exanthem subitum. No pathogens were identified in six patients in the PEE group and 11 in the non-PEE group.

ASMs were started in most cases with CBZ (*n* = 20) or LEV (*n* = 19). These medications were administered orally or through a nasogastric tube at first, and later changed to oral administration without a loading dose. Two of the 20 patients treated with CBZ developed a rash after 7–10 d, requiring a switch to topiramate or zonisamide. Valproic acid (VPA) was started in one patient, and no ASMs were initiated in five patients. The univariate analysis between the two groups found statistical significance in GCS 12–24 h, the number of patients with AESD with biphasic course, Tada score, and PCPC at 6 months after the onset (*p* = 0.006, 0.033, 0.002, 0.004, respectively, Supplementary Figures 5–7).

### 3.3 Multivariate logistic analysis for PEE in patients with AESD

GCS 12–24 h was excluded because the Tada score includes GCS 12–24 h as one of the seven components of the score.

The Late-Hypo group had more patients with AESD with a biphasic course than the Non-Hypo group (*p* = 0.005), reflecting the indication of therapeutic hypothermia between July 2008 and December 2020. Furthermore, there was a confounding factor that existed between the biphasic course and the treatment options of the Early-Hypo, Late-Hypo, or Non-Hypo groups. Patients with AESD in the Early-Hypo group do not have a biphasic course naturally because they underwent intravenous anesthesia with therapeutic hypothermia, masking the biphasic course. Consequently, the biphasic course was excluded from further multivariate analysis.

To avoid multicollinearity, each association was examined between two variables with *p* < 0.05 in the univariate analysis of both groups, including the Tada score, and PCPC at 6 months after onset, alongside the treatment options of Early-Hypo, Late-Hypo, or Non-Hypo group. The Spearman’s rank correlation coefficient was used to evaluate associations between Tada scores and PCPC at 6 months after onset.

Significant associations were found: Tada score and PCPC at 6 months after onset (*r* = 0.481, *p* = 0.001); the treatment options (Early-Hypo, Late-Hypo, or Non-Hypo group) and Tada score (*p* = 0.002, Table 1). Finally, statistical significance was adjusted using the Benjamini–Hochberg procedure to correct for three multiple comparisons, with a false discovery rate of 0.05, and all three associations were considered significant (*p* < 0.05).

Therefore, GCS 12–24 h, 3 h, biphasic course, and PCPC at 6 months after onset were excluded from the multivariate logistic regression analysis. Lastly, the Tada score and the treatment options (Early-Hypo, Late-Hypo, or Non-Hypo), which were the main focus of this study, were included in the multivariate logistic analysis with backwards stepwise elimination of non-significant variables. The analysis identified that the Tada score as one of the independent variables associated with an increased risk of PEE (*p* = 0.005, Table 3) and the treatment options of Early-Hypo or Late-Hypo significantly reduced the occurrence of PEE (*p* = 0.028, 0.025, respectively), where one-sided test was conducted due to the previous report by Hoshide et al. on the effectiveness of therapeutic hypothermia on PEE (13).

**Table 3:** The result of multivariate logistic analysis.

	Odds ratio for PEE	(95% Confidence interval)	*p*-value
Tada score	2.730	(1.358–5.488)	0.003*
Early-Hypo – Non-Hypo	0.061	(0.003–1.062)	0.028*
Late-Hypo – Non-Hypo	0.086	(0.007–0.990)	0.025*

*p < 0.05, one-sided test was conducted due to the previous report on the effectiveness of therapeutic hypothermia on post-encephalitic epilepsy (PEE).

### 3.4 Characteristics of patients in the PEE group

**Figure 2:**
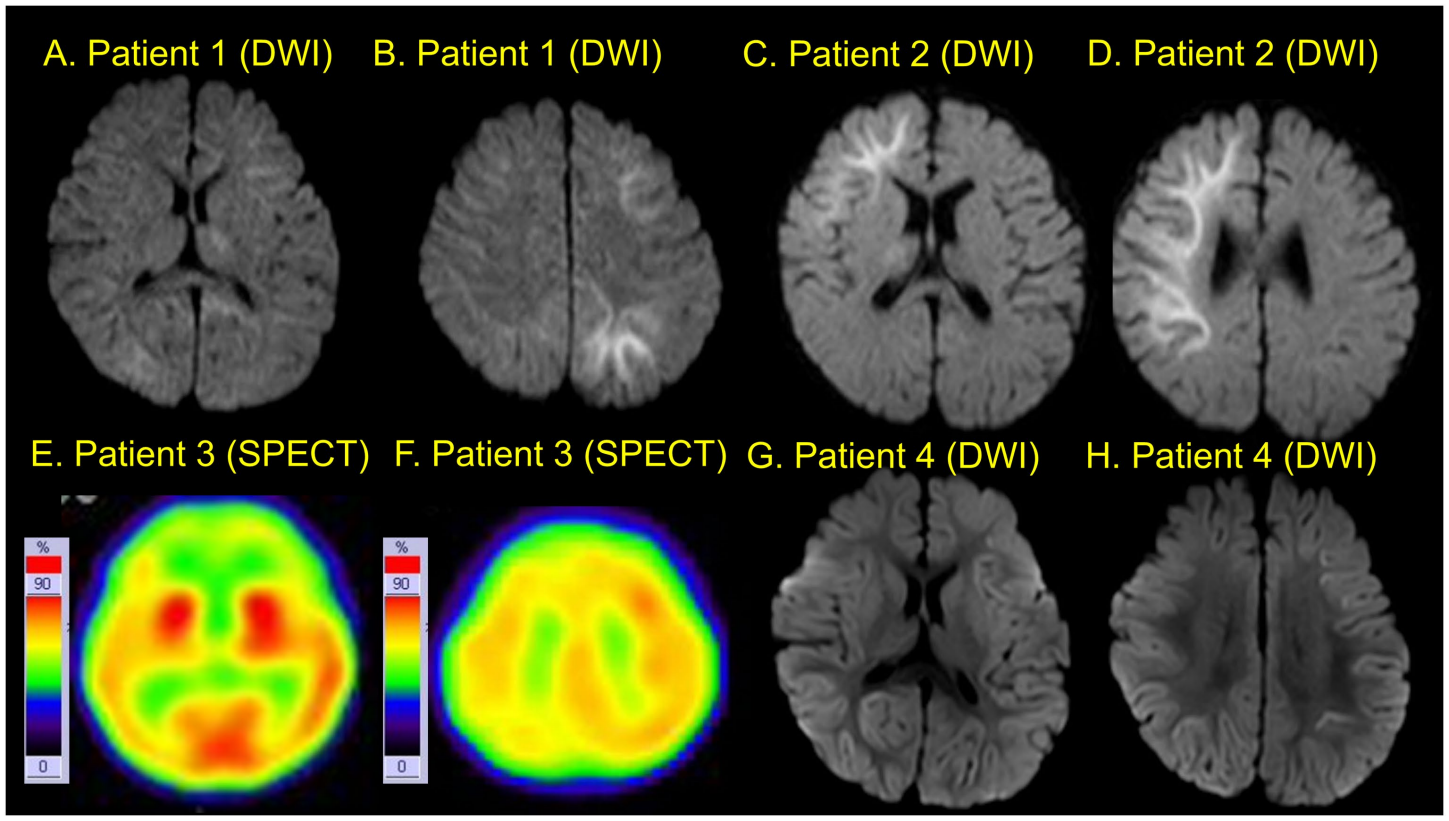
Neuroimaging (Patients 1–4). Two different slices of diffusion-weighted images **(A–D,G,H)** and SPECT images **(E,F)** for each patient. Patient 1 shows left frontal and bilateral parietal and occipital lesions with central sparing and a left thalamus lesion **(A,B)**. Patient 2 shows lesions in the right hemisphere without central sparing **(C,D)**. Patient 3 shows reduced blood flow in the right hemisphere on SPECT (using 99mTc-ECD) on day 15 after ESs **(E,F)**. Patient 4 shows left frontal and bilateral parietal lesions with central sparing **(G,H)**.

**Figure 3:**
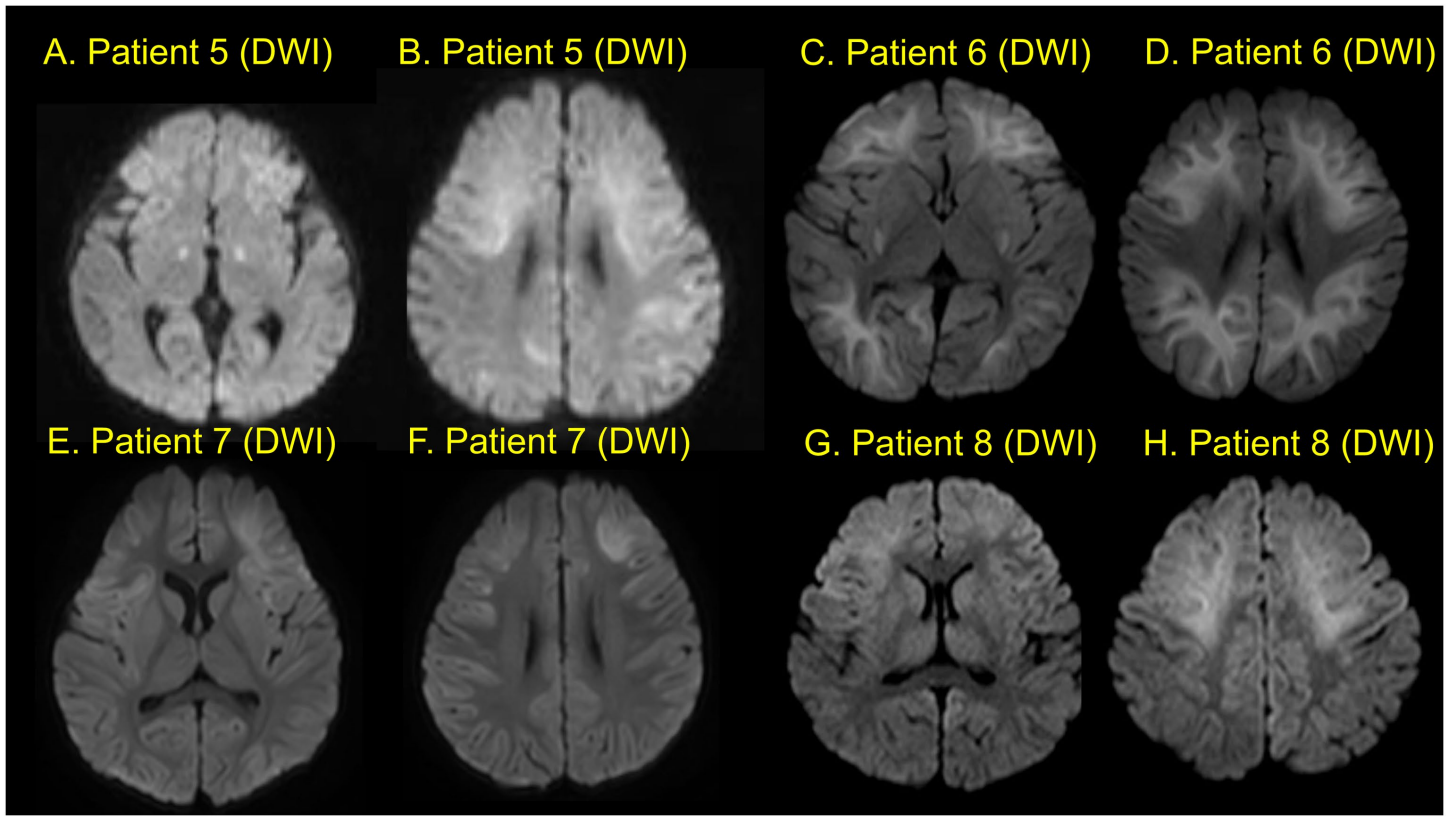
Neuroimaging (Patients 5–8). Two different slices of diffusion-weighted images **(A–H)** for each patient. Patient 5 shows bilateral frontal and parietal lesions with central sparing and bilateral basal ganglia lesions **(A,B)**. Patient 6shows lesions in the bilateral hemispheres with central sparing and bilateral basal ganglia lesions **(C,D)**. Patient 7 shows bilateral frontal lesions with central sparing **(E,F)**. Patient 8 shows bilateral frontal lesions with central sparing **(G,H)**.

**Figure 4:**
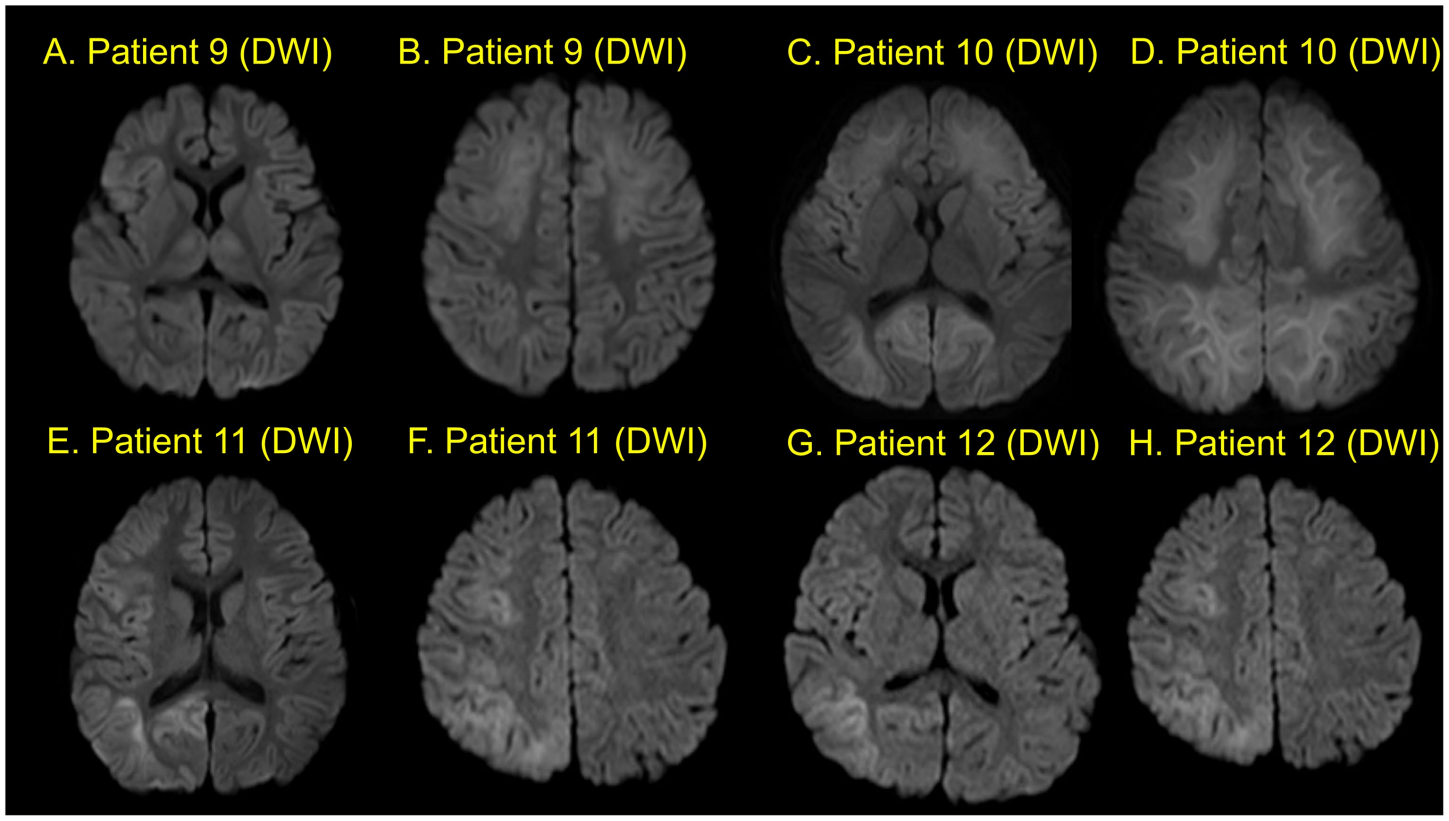
Neuroimaging (Patients 9–12). Patient 9 shows bilateral frontal and parietal lesions with central sparing and bilateral thalamus lesions **(A,B)**. Patient 10 shows lesions in both hemispheres with central sparing **(C,D)**. Patient 11 shows lesions in the right hemisphere with central sparing **(E,F)**. Patient 12 shows lesions in the right frontal, parietal, and occipital lobes with central sparing **(G,H)**.

The time interval between acute encephalopathy and the development of PEE was a median of 7 months (IQR, 1–15 months, range, 0.53–49 months). Regarding the classification of seizure types in PEE, eight patients experienced focal seizures, six patients had generalized seizures (myoclonic seizure, atypical absence seizure, generalized tonic seizures (GTS) or generalized tonic–clonic seizures GTCS), and two patients had both focal and GTS. Four out of five patients with basal ganglia and/or thalamus lesions experienced generalized seizures (GTS, GTCS, myoclonic seizure, or atypical absence seizures). Conversely, four patients without basal ganglia and/or thalamus lesions experienced only focal seizures during the observation period. Neuroimages of each patient are shown in Figures 2–4. Characteristics of each patient in the non-PEE group were also shown in Table 4.

**Table 4:** Characteristics of patients in the PEE group.

	Age at AESD onset (year, month)	Interval between AESD and PEE (months)	Treatment groups based on the timing of therapeutic hypothermia Early-Hypo/Late-Hypo/Non-Hypo	Sz type	Electroencephalography (interictal) around the time of PEE occurrence	ASM	ASM at the last follow-up	Thal/BG lesions	Lesions in the acute phase	Central sparing	Observation period	Sz frequency at the last follow-up	PCPC at 6 months	Neurodevelopment or cognitive test at follow-up (age, year, month)
1	7 m	9	Late	Focal Sz, GTS	Focal sharp wave	CBZ → ZNS, CZP, VPA → LEV, TPM	LEV	Lt Thal	Lt F, bil P, O	+	14y8m	Daily	3	FSIQ51 (WISC-IV) 14y10 m
2	7 m	0.57	Late	GTCS	Multifocal spike	CBZ → ZNS, CBZ, VPA	VPA	Rt Thal	Rt hemisphere	−	7y6m	None	4	−
3	10 m	1	Early	Focal Sz	No paroxysmal discharge	LEV	None	N/A	Rt hemisphere*	N/A	3y0m	None	2	DQ63 (KSPD) 3y4m
4	11 m	0.7	Early	Focal Sz	Focal spike	CBZ → CLB + VPA	CLB, VPA	none	Lt F, Bil P	+	2y2m	None	2	DQ71 (KSPD) 2y5m
5	1y2m	12	Non	Focal Sz	Focal spike	LEV→CBZ	CBZ	Bil BG	Bil F, P	+	7y4m	None	3	DQ32 (KSPD) 5y9m
6	1y3m	15	Late	Myoclonic Sz, atypical absence Sz, GTS	Diffuse slow spike and wave	LEV→VPA, CLB, PER	VPA, CLB, PER	Bil BG	Bil hemisphere	+	7y1m	Daily	3	DQ27 (KSPD) 4y5m
7	1y3m	30	Early	Focal Sz	No paroxysmal discharge	LEV	VPA, PER	None	Bil F	+	3y0m	None	2	DQ58 (KSPD) 3y6m
8	1y8m	3	Early	Focal Sz	−	LEV	LEV	None	Bil F	+	5y3m	Monthly	4	DQ31 (KSPD) 6y4m
9	1y8m	1	Late	Myoclonic Sz	Diffuse slow spike and wave	LEV→LEV, CLB, VPA, PER	CLB, VPA, PER	Bil Thal	Bil F, P	+	3y2m	None	4	DQ36 (KSPD) 4y0m
10	3y3m	5	Non	Myoclonic Sz	Multifocal spike	LEV	None	None	Bil hemisphere	+	8y0m	None	3	DQ70 (KSPD) 3y6m
11	6y0m	15	Non	Focal Sz	Focal spike	LEV	LEV	None	Lt hemisphere	+	7y3m	None	3	FSIQ82 (WISC-IV) 11y11m
12	8y0m	49	Non	Myoclonic, focal Sz	No paroxysmal discharge Abnormal background activity	CBZ	CBZ	None	Lt F, P, O	+	10y0m	Monthly	2	FSIQ82 (WISC-IV) 14y5m

*MRI was not performed in the acute phase due to the patient’s critical condition. SPECT (using 99mTc-ECD) showed reduced blood flow in the left hemisphere on day 15 after ES/ESs.

N/A, not applicable.

Sz, seizure; LEV, levetiracetam; CBZ, carbamazepine; VPA, valproic acid; ZNS, zonisamide; CLB, clobazam; PER, perampanel; Thal, thalamus; BG, basal ganglia; GTS, generalized tonic seizure; GTCS, generalized tonic -clonic seizure; Lt, left; Rt, right; F, frontal; Bil, bilateral; P, parietal; O, occipital; PCPC, the Pediatric Cerebral Performance Category; FSIQ, full scale intelligence quotient; WISC-IV, the Wechsler Intelligence Scale for Children Fourth Edition; DQ, development quotient; KSPD, the Kyoto Scale of Psychological Development.

## 4 Discussion

In this study, we examined the incidence of PEE and investigated whether therapeutic hypothermia reduced the risk of PEE in patients with AESD. The multivariate logistic analysis identified a higher Tada score as an independent variable associated with an increased risk of PEE, and either of the treatment options, Early-Hypo or Late-Hypo, significantly reduced the occurrence of PEE (Table 4).

In our present study, the prevalence of PEE in patients with AESD was 26.7% (12 out of 45), consistent with that reported by Ito et al., where the prevalence was 22.7% (10 out of 44 patients). Ichimiya et al. reported a higher prevalence of 45.5% (10 out of 22 patients), with at least four patients receiving therapeutic hypothermia. Similarly, Nishioka et al. reported a prevalence of 30% (6 out of 20 patients), where 19 patients underwent targeted temperature management. The difference between these studies may be attributed to variations in sample sizes and treatment protocols. The time interval between acute encephalopathy and PEE development in our study was a median of 7 months (IQR, 1–5 months; range, 0.53–49 months), consistent with the results of previous reports, medians of 2–14 months (range, 0.8–48 months) (10–13). According to Ito’s report, 9 out of 10 patients developed PEE within 18 months (11), and in Ichimiya’s report, all patients developed PEE within a year (10). These findings further suggest that our results align with previous studies.

The univariate analysis between the PEE and non-PEE groups showed statistical significance in GCS 12–24 h, the number of patients with AESD with biphasic course, Tada score, and PCPC at 6 months after onset.

We observed that Time_1st-cooling_ strongly correlated with Time_1st-2nd_ as patients were predominantly diagnosed after the emergence of LSs and underwent therapeutic hypothermia (6). This correlation analysis indicated that the later therapeutic hypothermia was initiated in patients with AESD, the better the outcomes appeared, likely introducing bias. To mitigate this, we used the treatment options of Early-Hypo, Late-Hypo, or Non-Hypo, based on whether therapeutic hypothermia was initiated before or after the appearance of LSs. The analysis revealed that the treatment options of Early-Hypo or Late-Hypo significantly reduced the occurrence of PEE. Additionally, a higher Tada score was a significant risk factor for PEE development.

Whether therapeutic hypothermia was initiated before or after LSs, it reduced the occurrence of PEE in our study. However, there is still a lack of direct evidence that therapeutic Hypothermia can improve the outcome of AESD patients, except for the indirect beneficial evidence reported by Sakata et al. that the earlier therapeutic hypothermia is initiated, the better the outcome (6).

Nishioka et al. reported that patients in the PEE group had higher serum levels of AST and ALT at the onset of AESD and experienced longer ES duration (12). Similarly, Ichimiya et al. reported that patients in the PEE group had slightly longer ES durations and more frequently exhibited coma or involuntary movements during the course of AESD compared to those in the non-PEE group (10). While there was no significant difference between the PEE and non-PEE groups in AST, ALT, or ES duration in our study, our findings indicated that the Tada score, which incorporates AST levels of 40 U/L or higher and ES durations of 40 min or longer among its six components, is predictive of PEE development. We did not examine involuntary movements due to the difficulty of distinguishing between AESD-related movements and sedation withdrawal symptoms, particularly given the use of sedation during and after therapeutic hypothermia. The Tada score may better capture the overall severity of AESD after ESs and more accurately predict PEE development than individual factors like AST, ALT, or ES duration. Additionally, our earlier study demonstrated that the Tada score is not only predictive of AESD development but also associated with worse outcomes (6).

Four out of five patients with basal ganglia and/or thalamus lesions experienced generalized seizures (GTS, GTCS, myoclonic seizures, or atypical absence seizures), while those without these lesions had focal seizures during the observation period. Additionally, patients in the PEE group tend to have thalamus and/or basal ganglia lesions more frequently than those in the non-PEE group, although the difference was not statistically significant. Substantial evidence suggests that thalamocortical circuits are involved in generalized epilepsy and that the basal ganglia contribute to various forms of seizures (22, 23). Furthermore, Sakata et al. reported that basal ganglia and/or thalamus lesions exacerbated outcomes measured by PCPC (6). Similarly, Ito et al. speculated that abnormal neuronal networks between the cortex and subcortical structures may form during the recovery from diffuse white matter damage in AESD, potentially triggering epileptic spasms. They proposed that this mechanism mirrors how white matter maturation can trigger spasms in patients with infantile spasm, as diffuse subcortical white matter damage was observed in PEE patients in their study (11). The association between thalamus and/or basal ganglia lesions, seizure types, and therapeutic hypothermia in PEE requires further clarification in future prospective studies.

Beyond conducting the study at a single tertiary center and including a small sample size of patients with AESD, there are other limitations to our study. First, the observation period for one patient in our study was 12 months, which was shorter than in previous reports. Moreover, nine out of 10 patients developed PEE within 18 months, while in Ichimiya’s report (10, 11), all patients developed PEE within a year. Hence, this case was deleted from this study as described in the method section. Furthermore, in our study, the observation period for PEE was more than 20 months in all patients, which was considered appropriate. Second, there could have been additional cases of PEE if patients had not been given ASM prophylactically after the acute phase. In our study, PEE was defined as multiple seizures occurring after 14 d from the early seizure, requiring a dosage increment of ASM or the addition of a new ASM, irrespective of prophylactic use. However, previous reports by Ito et al. and Ichimiya et al. (9) revealed that eight out of 10 and seven out of 10 patients, respectively, required more than two ASMs. Thus, the potential underestimation of PEE occurrence in this study was considered minimal. Further, which ASM used after the acute phase reduce the occurrence of PEE was out of the scope of this study and requires future RCT trials in AESD patients.

Although a future prospective study involving a larger number of patients is required, the current study revealed that a higher Tada score is a risk factor for developing PEE among patients with AESD who underwent therapeutic hypothermia. Moreover, therapeutic hypothermia reduced the risk of PEE, regardless of whether it was initiated before or after LSs.

## Data Availability

The raw data supporting the conclusions of this article will be made available by the authors, without undue reservation.

## Footnotes

^1^ http://R-project.org/

## Glossary

AESD: acute encephalopathy with biphasic seizures and late reduced diffusion
ES: early seizure
LSs: late seizures
Sz: seizure
DWI: diffusion-weighted imaging
PEE: post-encephalitic epilepsy
MRI: magnetic resonance imaging
SPECT: single photon emission computed tomography
RIPoC: remote ischaemic postconditioning
AST: aspartate transaminase
ALT: alanine transaminase
ASM: antiseizure medication
LEV: levetiracetam
CBZ: carbamazepine
VPA: valproic acid
ZNS: zonisamide
CLB: clobazam
PER: perampanel
Thal: thalamus
BG: basal ganglia
GTS: generalized tonic seizure
GTCS: generalized tonic–clonic seizure
