# Supplementary material for: Post-encephalitic epilepsy in patients with acute encephalopathy with biphasic seizures and late reduced diffusion
Source: Front Neurol. 2025 Jul 22;16:1568566. doi: 10.3389/fneur.2025.1568566 (PMC12321538; doi:10.3389/fneur.2025.1568566)
Supplement: Supplementary file 1 [file Table_1.docx]

| Supplementary Table 1: Multiple comparisons among patients in the Early-Hypo, Late-Hypo, and Non-Hypo groups (n = 45) | | | | | | | | | | |
| --- | --- | --- | --- | --- | --- | --- | --- | --- | --- | --- |
|  | Early-Hypo group | | | Late-Hypo group | | | Non-Hypo group | | | p value |
|  | n = 11 | | | n = 24 | | | n = 10 | | |  |
| Number of patients with AESD with PEE in whom ASM were terminated, n (%) | 1 | (9.1) | 11 | 0 | (0) | 24 | 1 | (10) | 10 | 0.709 |
| Duration of ASM medication in patients with AESD for whom ASM was terminated in months, median (IQR), n | 33 | (26.5-35.5) | 7 | 27 | (27–30) | 17 | 17.5 | (11.5-27) | 4 | 0.316 |
| Duration of ASM medication in patients with AESD in the PEE group who are still on ASM at the time of investigation in months, median (IQR), n | 36 | (32-49.5) | 3 | 90 | (7163.5-137.5) | 4 | 88 | (87.5-104) | 3 | 0.092 |
| Observation period in months, median (IQR), n | 79 | (36.5-103) | 11 | 67.5 | (36.5-94) | 24 | 87.5 | (74-117) | 10 | 0.495 |

AESD, acute encephalopathy with biphasic seizures and late reduced diffusion; PEE, post-encephalitic epilepsy; ASM, antiseizure medication.
