# Supplementary material for: Post-encephalitic epilepsy in patients with acute encephalopathy with biphasic seizures and late reduced diffusion
Source: Front Neurol. 2025 Jul 22;16:1568566. doi: 10.3389/fneur.2025.1568566 (PMC12321538; doi:10.3389/fneur.2025.1568566)
Supplement: Supplementary file 2 [file Table_2.docx]

| Supplementary Table 2: Univariate analysis between PPE and non-PEE groups (n = 45) | | | | | | | |
| --- | --- | --- | --- | --- | --- | --- | --- |
|  | PEE group | | | Non-PEE group | | | p value |
|  | n = 12 | | | n = 33 | | |  |
| Prophylactic use of ASM, n (%) | 12 | (100) | 12 | 28 |  | 33 | 0.303 |
| First ASM used after the acute phase |  |  |  |  |  |  | 0.063 |
| CBZ, n (%) | 3 | (25) |  | 15 | (45.5) |  |  |
| LEV, n (%) | 9 | (75) |  | 10 | (30.3) |  |  |
| Others (TPM, ZNS, or VPA), n (%) | 0 | (0) |  | 3 | (9.0) |  |  |
| None, n (%) | 0 | (0) |  | 5 | (15.2) |  |  |
| Duration from the onset of AESD to the development of PEE, in months, median (IQR), n | 7 | (1–15) | 12 | N/A | N/A | N/A | - |
| Number of patients with AESD with PEE in whom ASM were terminated, n (%) | 2 | (16.7) | 12 | N/A | N/A | N/A | - |
| Duration of ASM medication in patients with AESD for whom ASM was terminated, in months, median (IQR), n | 31 | (26–36) | 2 | 27 | (18–34) | 26 | 0.825 |
| Duration of ASM medication in patients with AESD who are still on ASM, in months, median (IQR), n | 87 | (40–93) | 10 | 81.5 | (71–92) | 2 | 1.000 |
| Observation period in months, median (IQR), n | 87 | (38–94.5) | 12 | 74 | (37–108) | 33 | 0.970 |

LEV, levetiracetam; CBZ, carbamazepine; VPA, valproic acid; ZNS, zonisamide;

N/A: not applicable
