# Supplementary material for: Post-encephalitic epilepsy in patients with acute encephalopathy with biphasic seizures and late reduced diffusion
Source: Front Neurol. 2025 Jul 22;16:1568566. doi: 10.3389/fneur.2025.1568566 (PMC12321538; doi:10.3389/fneur.2025.1568566)
Supplement: Supplementary file 3 [file Presentation_1.pptx]

## Slide 1
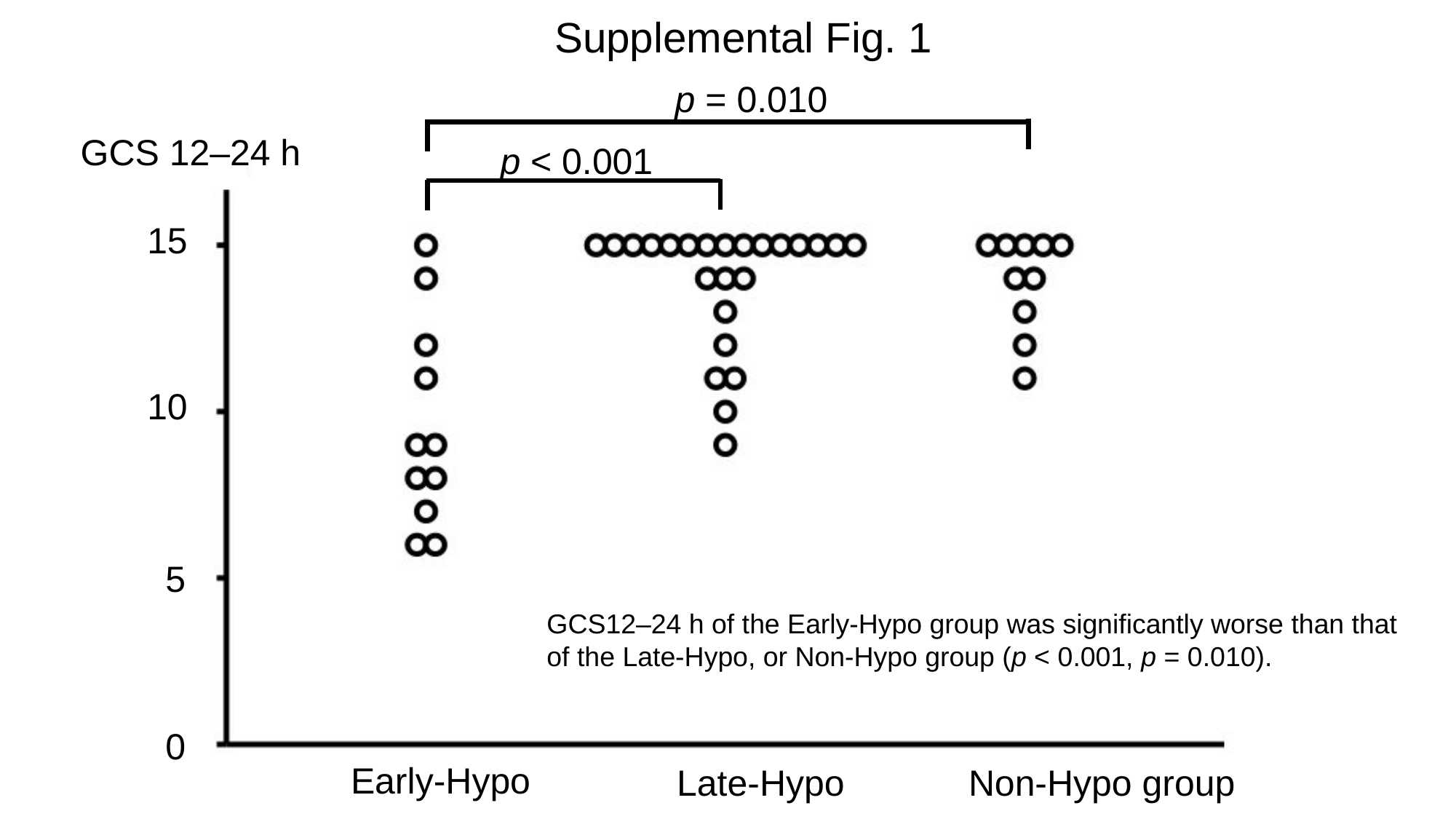

Supplemental Fig. 1
p = 0.010
GCS 12–24 h
p < 0.001
15
10
5
GCS12–24 h of the Early-Hypo group was significantly worse than that
of the Late-Hypo, or Non-Hypo group (p < 0.001, p = 0.010).
0
Early-Hypo
Late-Hypo
Non-Hypo group

## Slide 2
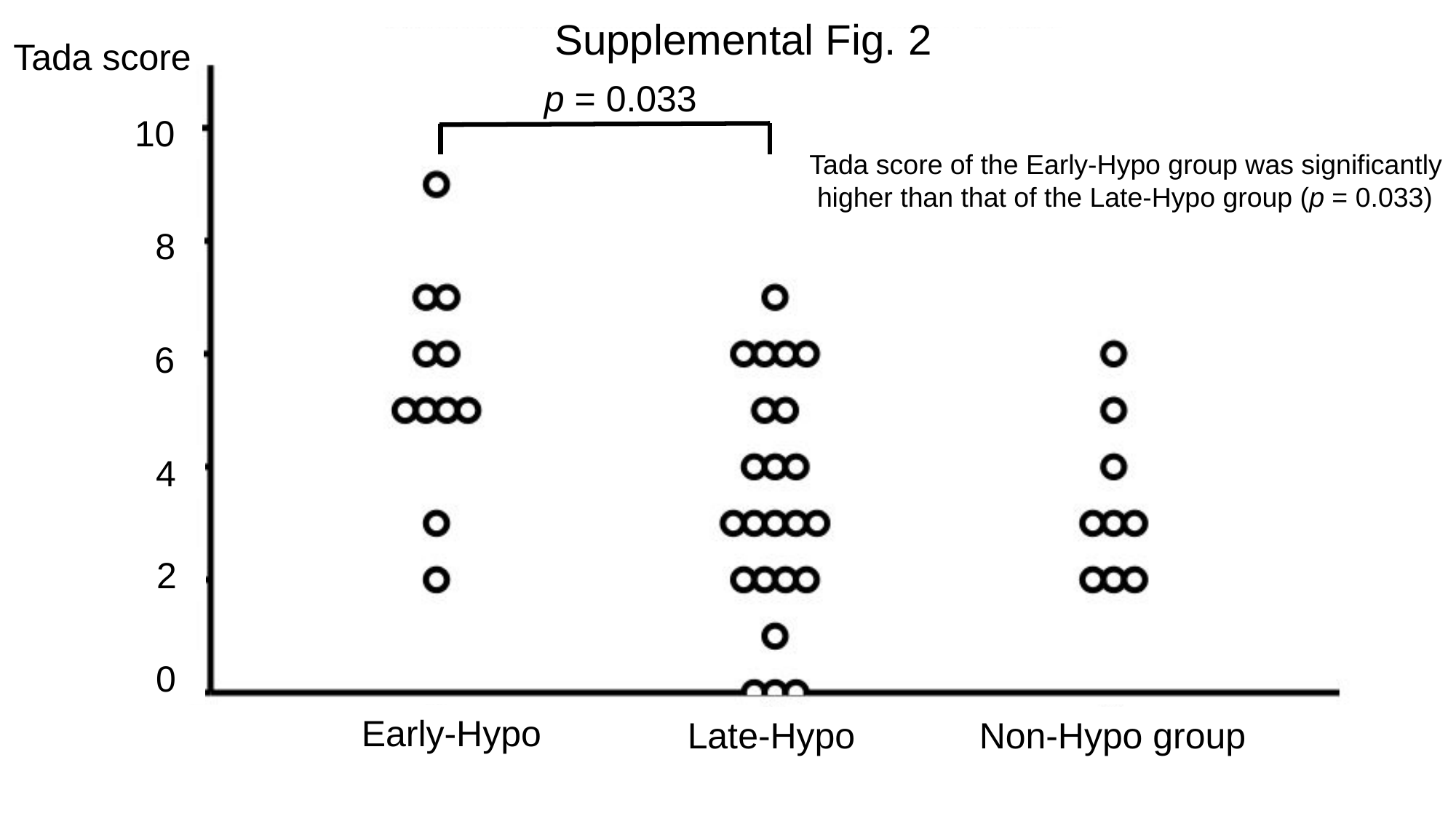

Supplemental Fig. 2
Tada score
p = 0.033
10
Tada score of the Early-Hypo group was significantly
 higher than that of the Late-Hypo group (p = 0.033)
8
6
4
2
0
Early-Hypo
Late-Hypo
Non-Hypo group

## Slide 3
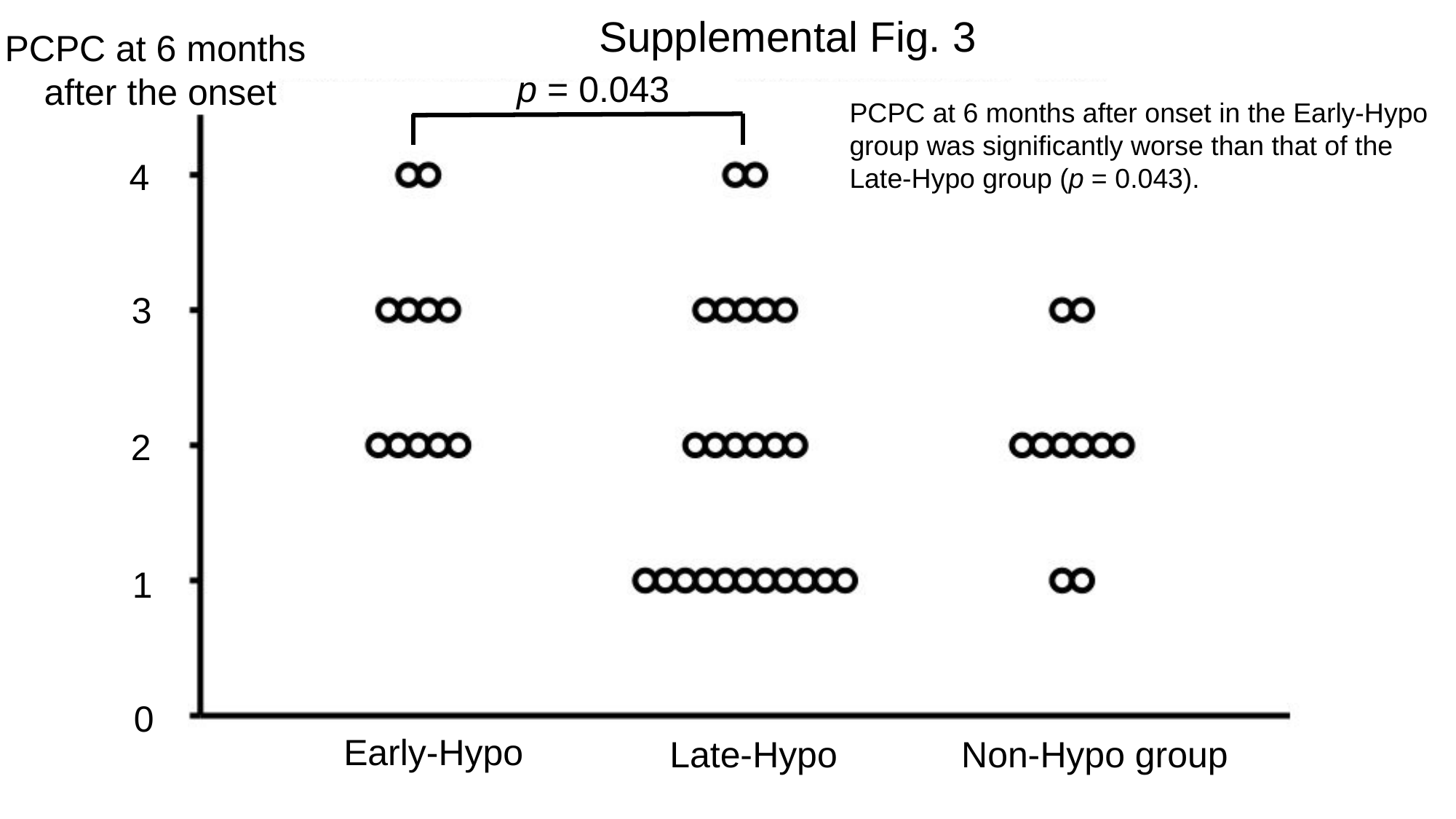

Supplemental Fig. 3
PCPC at 6 months
after the onset
p = 0.043
PCPC at 6 months after onset in the Early-Hypo
group was significantly worse than that of the
Late-Hypo group (p = 0.043).
4
3
2
1
0
Early-Hypo
Late-Hypo
Non-Hypo group

## Slide 4
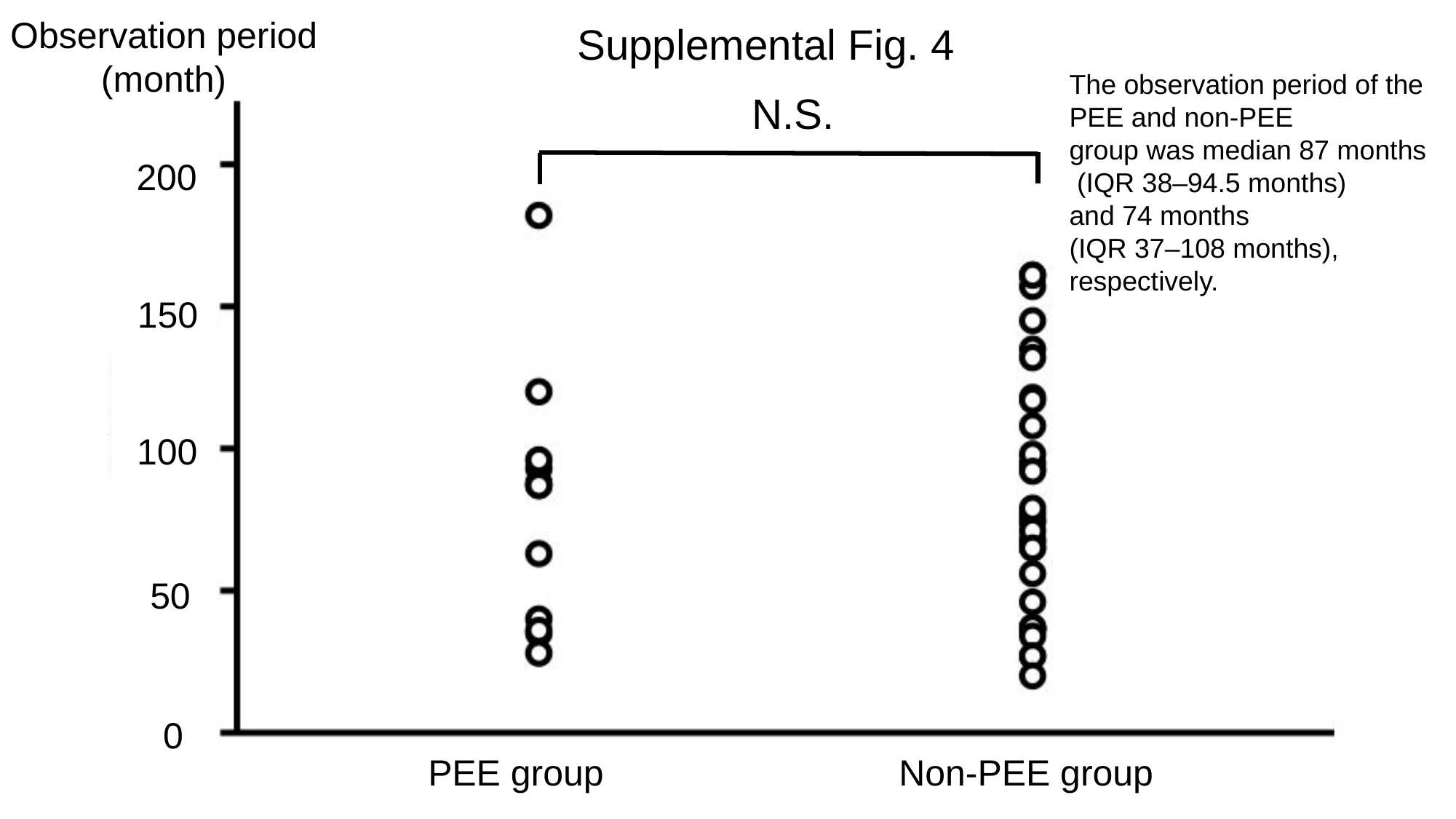

Observation period
(month)
Supplemental Fig. 4
The observation period of the
PEE and non-PEE
group was median 87 months
 (IQR 38–94.5 months)
and 74 months
(IQR 37–108 months),
respectively.
N.S.
200
150
100
50
0
PEE group
Non-PEE group

## Slide 5
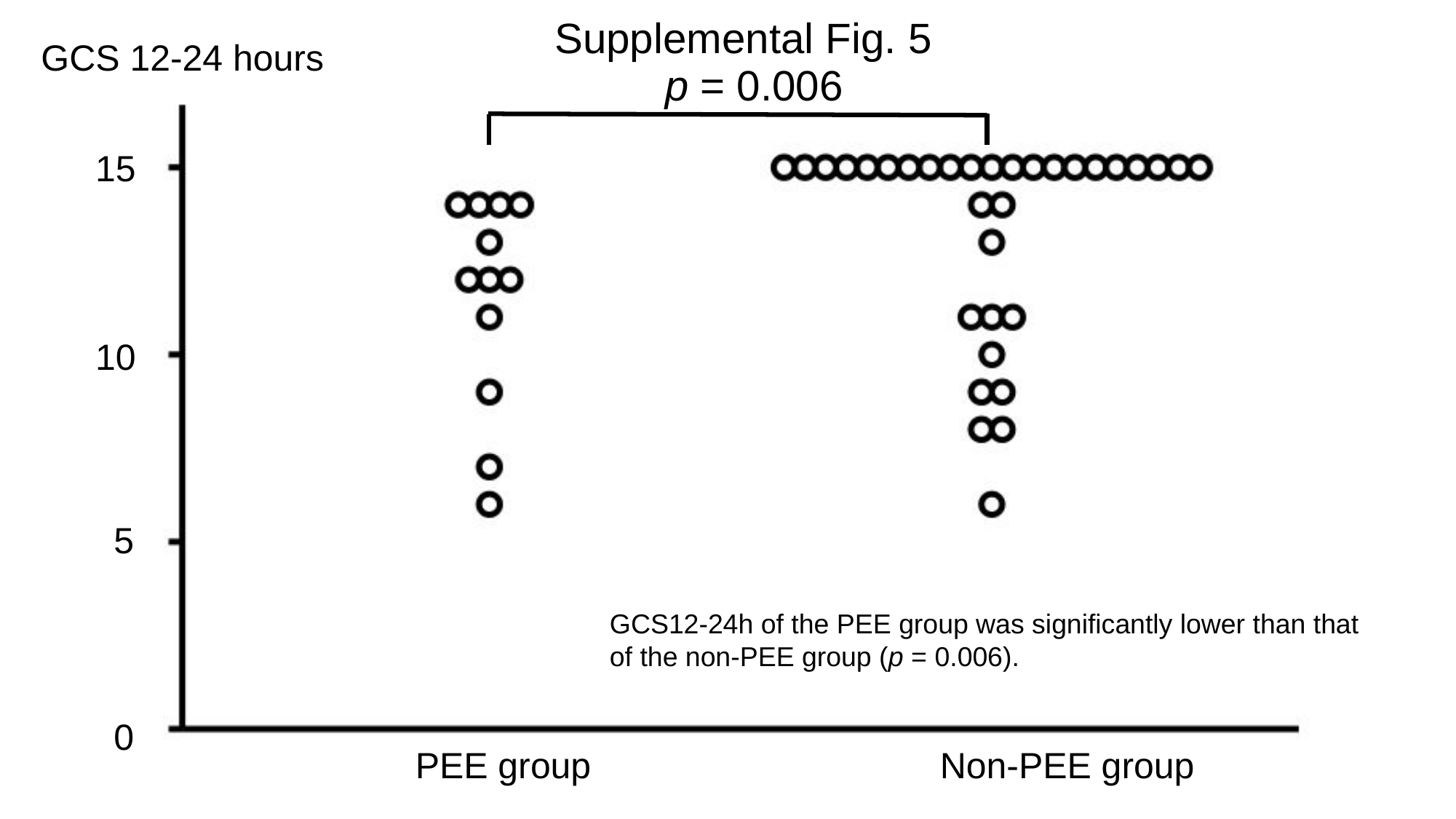

Supplemental Fig. 5
GCS 12-24 hours
p = 0.006
15
10
5
GCS12-24h of the PEE group was significantly lower than that
of the non-PEE group (p = 0.006).
0
PEE group
Non-PEE group

## Slide 6
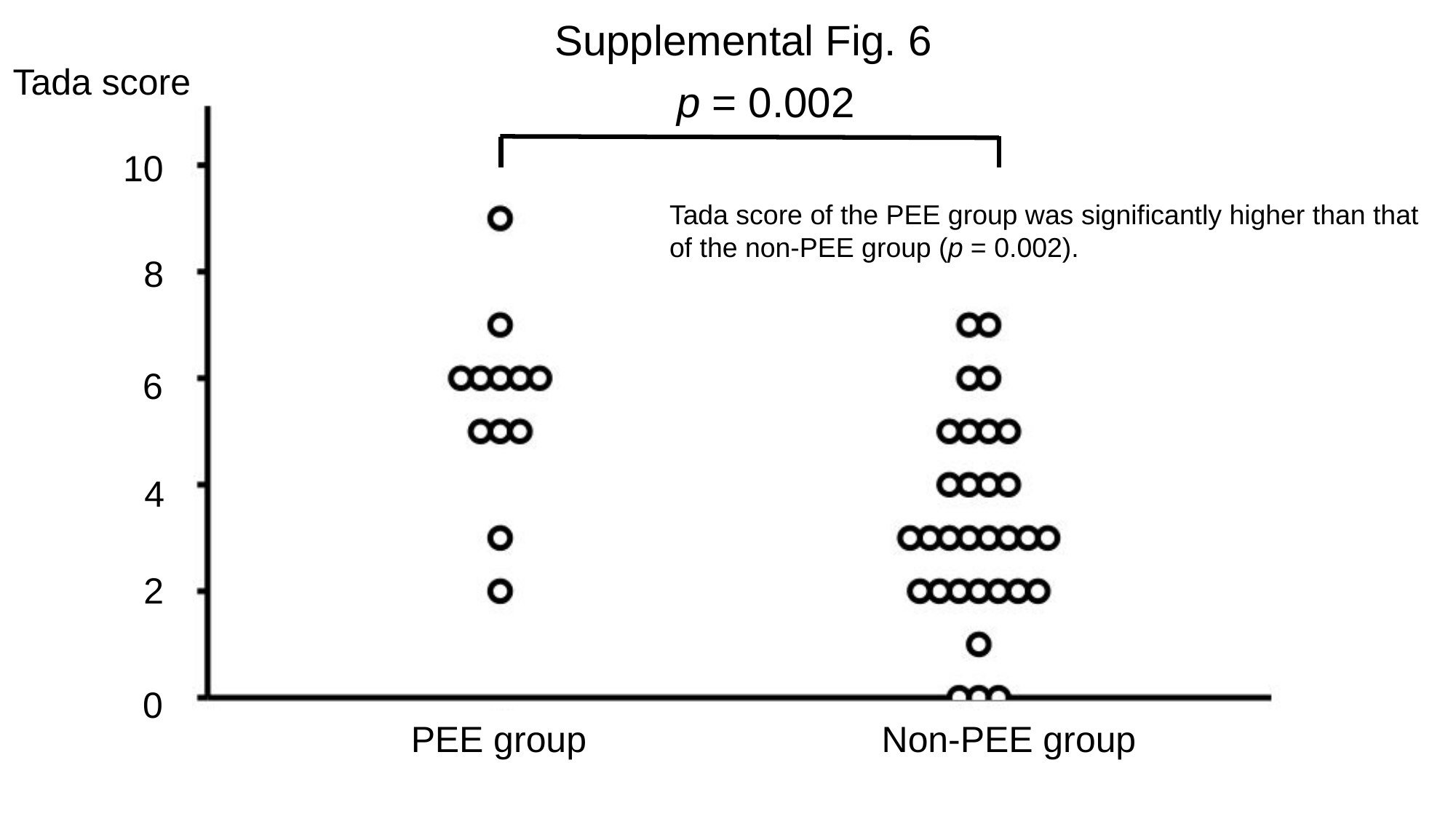

Supplemental Fig. 6
Tada score
p = 0.002
10
Tada score of the PEE group was significantly higher than that
of the non-PEE group (p = 0.002).
8
6
4
2
0
PEE group
Non-PEE group

## Slide 7
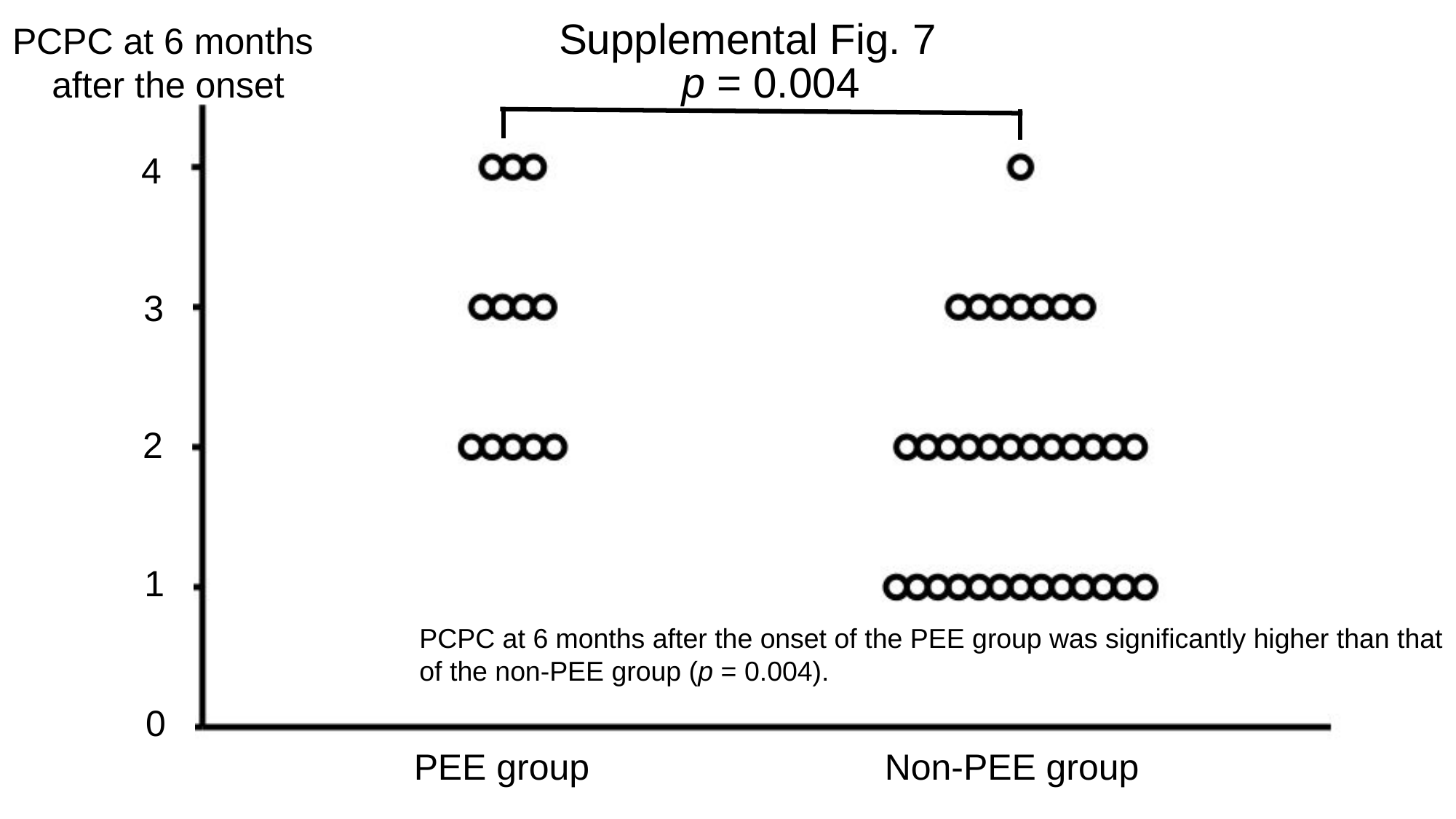

Supplemental Fig. 7
PCPC at 6 months
after the onset
p = 0.004
4
3
2
1
PCPC at 6 months after the onset of the PEE group was significantly higher than that
of the non-PEE group (p = 0.004).
0
PEE group
Non-PEE group
